# Loxosceles reclusa Envenomation Causing Acute Hemolytic Anemia: A Case Report on Loxoscelism

**DOI:** 10.7759/cureus.64413

**Published:** 2024-07-12

**Authors:** Steven J Laxton, David Whetstone

**Affiliations:** 1 Department of Emergency Medicine, University of Tennessee Health Science Center (UTHSC) Nashville - Saint Thomas Health, Murfreesboro, USA; 2 Department of Emergency Medicine, Ascension Health, Murfreesboro, USA

**Keywords:** toxicology and envenomation, loxocelism, s: anemia, dermal necrosis, autoimmune hemolytic anemia (aiha), brown recluse spider

## Abstract

This case report has the main objective of providing education surrounding the presentation, evaluation, diagnosis, and treatment of *Loxosceles reclusa* envenomation and presenting a case of loxoscelism that occurred in an adult that subsequently presented to the emergency department. A secondary objective of this case report is to add to the literature of images bite wound images associated with loxoscelism that resulted in inpatient admission and treatment for acute hemolytic anemia.

## Introduction

Approximately 1% of confirmed recluse bites have systemic effects [1]. This case is among the 1% as this patient developed acute hemolytic anemia necessitating transfusion. This case report also adds to the literature of loxoscelism with systemic effects as well as another image of a bite with both local and systemic symptoms including dermal necrosis and systemic effects of acute hemolytic anemia requiring inpatient admission for monitoring and transfusion. Of note, this article was previously posted to the medRxiv preprint server on June 12, 2024.

## Case presentation

**Figure 1:**
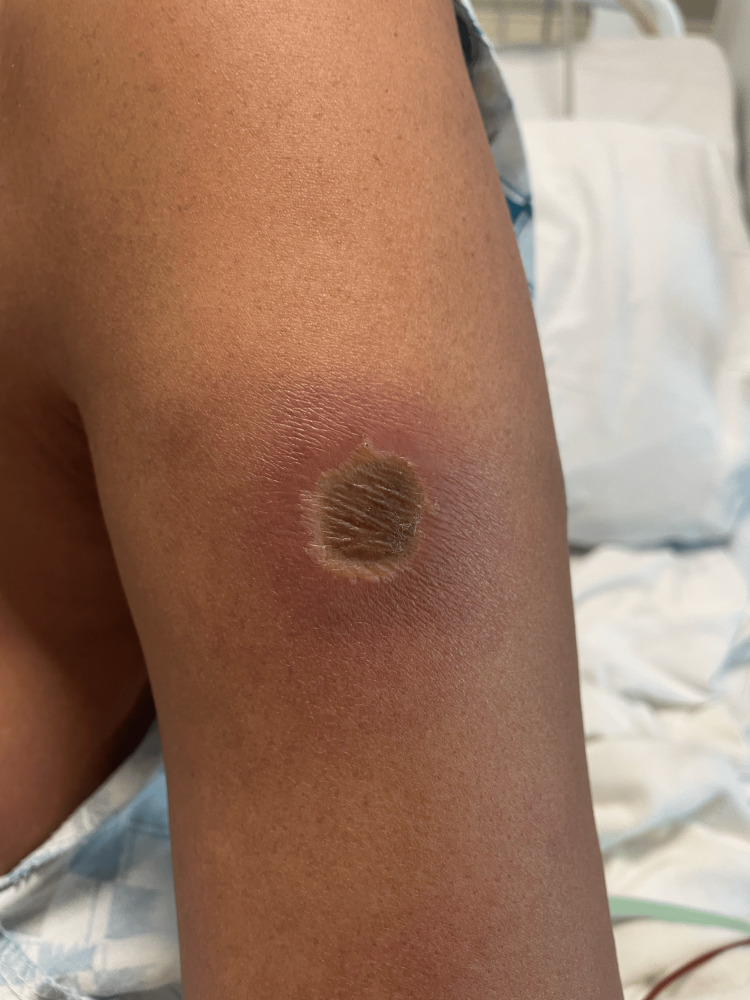
Wound on the left lateral mid-arm with necrotic core and surrounding erythema

This patient is a 41-year-old female with no significant past medical history who presented to the emergency department with concerns about a spider bite on her left upper extremity that occurred five days prior to presentation. She stated that she witnessed a spider crawling on her and the sensation of a “bite” in the area that then developed the finding of erythema surrounding an area of necrosis (Figure 1). She stated that she had seen brown recluses in her house. Other symptoms reported were diffuse itchiness and associated vomiting since being bitten. She demonstrated a wound from the bite on her left upper extremity, which gradually worsened, having started with a little blister at the bite wound that developed to an area of erythema with a necrotic core. 

Upon presentation, the patient had vital signs most concerning for tachycardia of 128 beats per minute and physical exam findings of an erythematous circular spot on the left lateral mid arm with a necrotic core. A small area of surrounding induration without fluctuance. It is important to remember that wound appearance and severity of envenomation do not correlate [2]. She also had mild jaundice and scleral icterus on examination.

A laboratory examination was performed and found to have significant abnormalities in serum electrolytes, hepatic function, and blood count. The electrolyte abnormality was hypokalemia which was measured at 2.6 mmol/L. The hepatic function panel showed a mild elevation in liver enzymes (AST 55 U/L) and a total bilirubin of 2.2 mg/dL. The blood counts showed an elevated white count (22.3 x 103/mm3) and a depressed red blood count (3.93 x 106/mm3) with an associated depressed hemoglobin (7.8 g/dL). Other laboratory measurements returned reassuring without elevation in creatinine kinase, international normalized ratio, prothrombin time, or partial thromboplastin time. 

The patient was treated with electrolyte replacement, IV fluids, and started on vancomycin and ceftriaxone for concern of possible secondary bacterial infection. The decision was then made to admit the patient given the concern for *Loxosceles reclusa *envenomation and anemia that would likely necessitate transfusion. 

**Figure 2:**
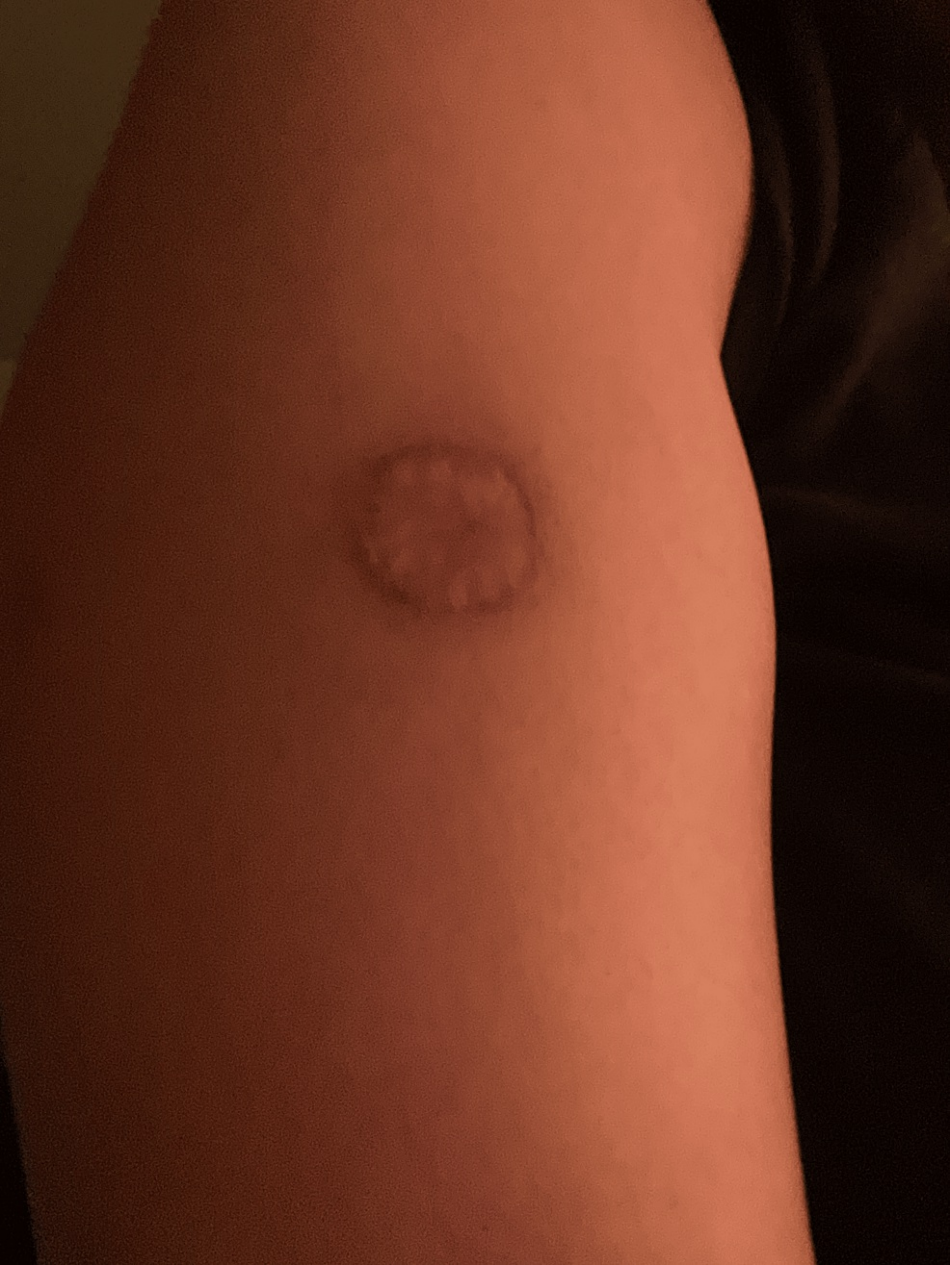
Healed area of dermal necrosis following recluse bite as of March 13, 2024

During inpatient admission, the patient’s hemoglobin continued to downtrend to 5.8 g/dL requiring 1 unit packed red blood cell transfusion to which the patient responded and did not need further transfusions. The patient’s hyperbilirubinemia resolved as well as electrolyte abnormalities. Two blood cultures grew no organisms. Antibiotic therapy of vancomycin and ceftriaxone was continued for three days until the decision was then made that the patient was stable enough for discharge home with amoxicillin-clavulanic acid and doxycycline for cellulitis on the arm surrounding the area of spider bite. The area of dermal necrosis also healed without the need for further intervention but did develop a notable scar as seen in Figure 2.

**Figure 3:**
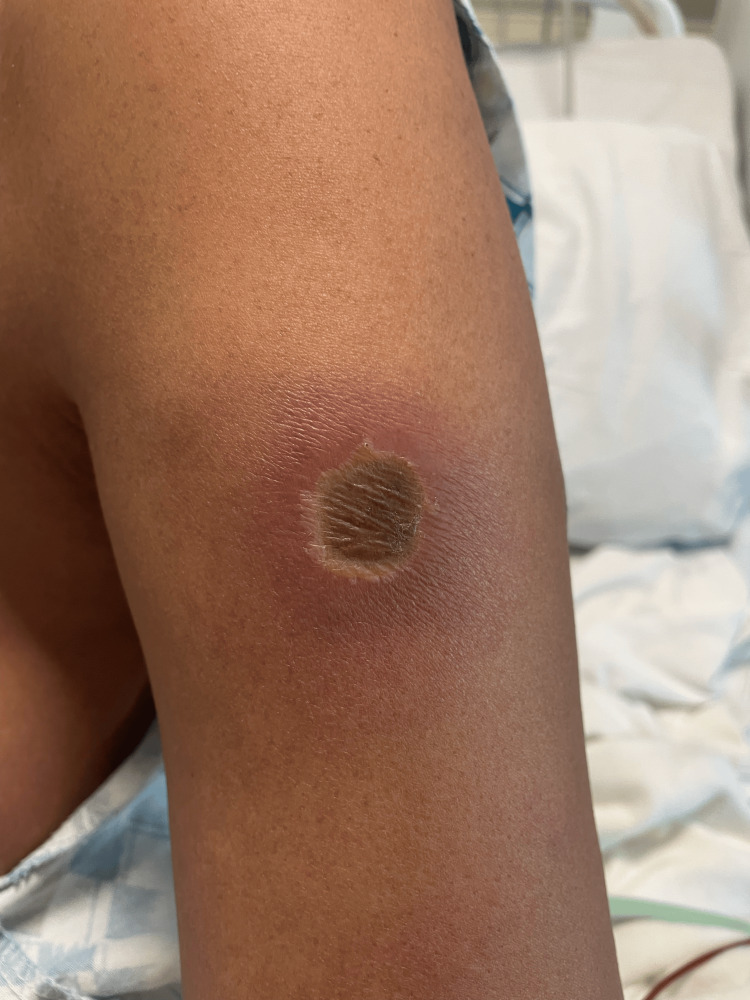
Area of recluse bite prior to presentation with start of necrosis

**Figure 4:**
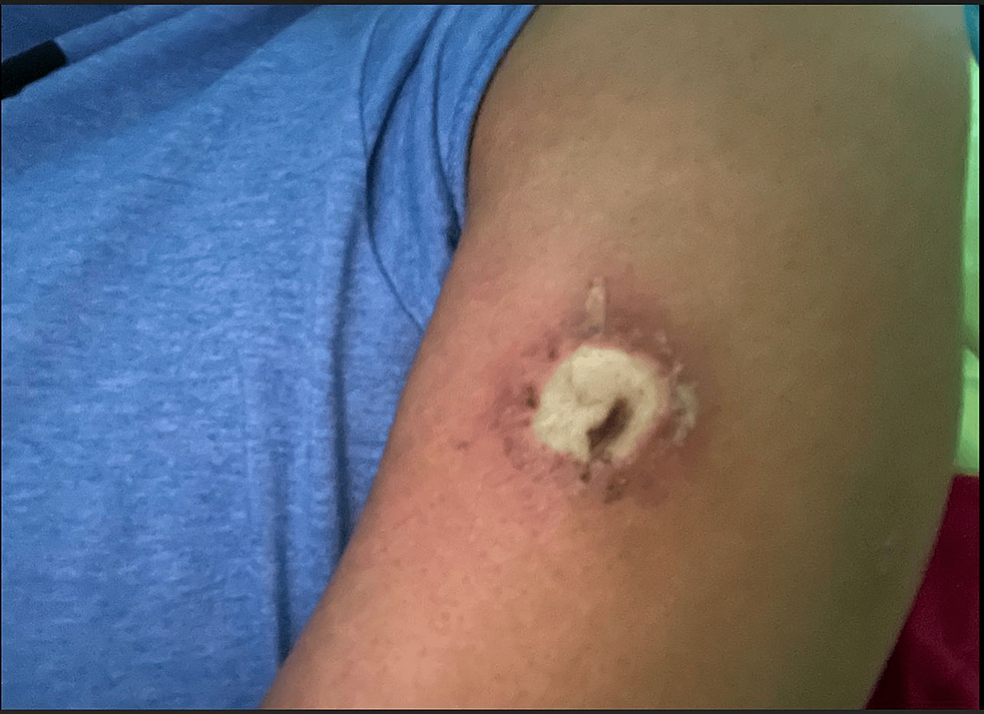
Area of recluse bite while hospitalized with continued necrosis and reactive skin changes versus secondary bacterial infection surrounding the bite

**Figure 5:**
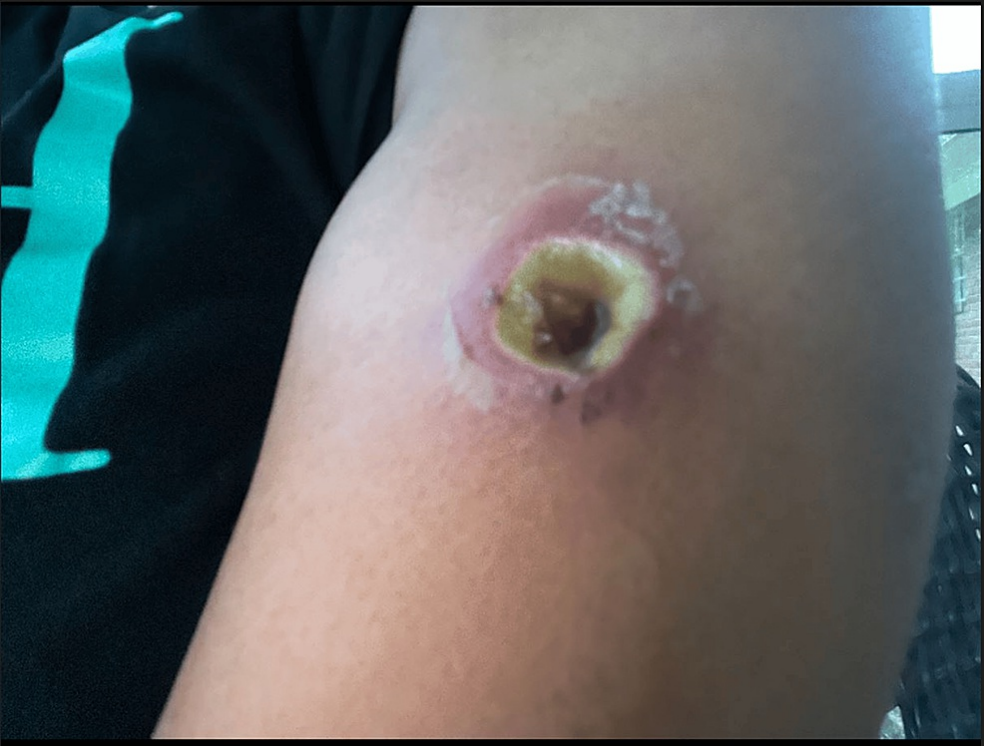
Further development of central necrosis with sloughing of necrotic skin

**Figure 6:**
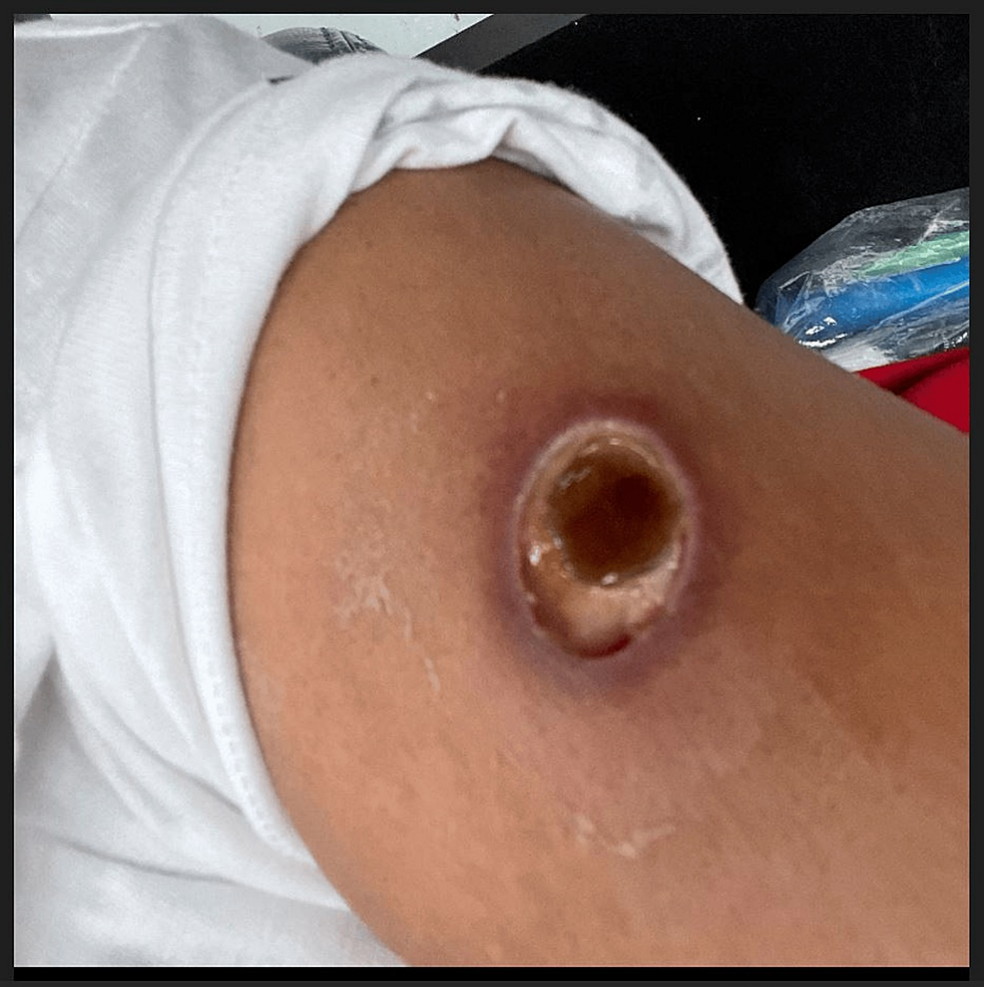
Development of liquefactive necrosis and sharp demarcation of normal skin at the wound edge

The patient also graciously provided further images of the bite wound that range from prior to presentation (Figure 3), while in the hospital (Figure 4) and the evolution of dermal necrosis following discharge to home (Figures 3-6). These images following discharge home provide a good example of the evolution of dermal necrosis from the start of central pallor to the necrotic core and the development of liquefactive necrosis. 

## Discussion

Brown recluse spiders (*Loxosceles reclusa*) are native spiders to North America (Appendix A) and are found in the mid-southern United States. Recluse spiders are commonly found in homes and basements [1] and are considered synanthropic spiders (i.e., their population numbers increase in association with humans). Within their endemic habitats, these spiders are commonly encountered within locations of homes not commonly used such as behind furniture, closets, basements, etc [2].

There are many methods for identification, however, the most accurate method of identifying a recluse spider involves counting the eyes if one is able to safely identify them by up close examination. Most spiders have eight eyes in two rows of four. In contrast, recluse spiders have six eyes, with a pair in front, a pair on both sides, and a gap between the pairs. With the naked eye or low magnification, the eye pairs (dyads) may appear as individual eyespots (Appendix B). A less reliable method for identification is via body markings. The brown recluse has a violin pattern on its anterior cephalothorax, but this method is not as reliable as the “violin pattern” varies with spider age [3].

Loxoscelism is the term for the medical manifestations of bites by recluse spiders which can include both local and systemic affects. 

The venom produced includes many enzymes that can cause injury but most notably, the phospholipases D (formerly designated as sphingomyelinases) are the culprit for causing most of the clinical entity of loxoscelism. This enzyme is the major culprit in causing necrotic loxoscelism, platelet disorders, hemolysis, and acute renal failure [4].

Local effects can vary from two small puncture wounds that do not draw attention to dermal and myodermal necrosis. Usually, the local effects are the development of a red plaque that later progresses to central pallor. The pain typically increases over the next two to eight hours and may become severe. It may develop a vasculitic appearance. In most cases, however, this lesion is self-limited and resolves without further complications in approximately one week. However, in some patients, the lesion can develop a dark, depressed center over the ensuing 24 to 48 hours (about two days), culminating in a dry eschar that subsequently ulcerates [5].

Systemic symptoms are an infrequent complication [6] of recluse bites that do not correlate with local findings. The main systemic symptoms include malaise, nausea and vomiting, fever, myalgias with dark urine (rhabdomyolysis), pallor, jaundice, icterus, and painless dark urine (acute hemolytic anemia) [5].

Rare and uncommon life-threatening complications following a recluse spider bite include angioedema, acute hemolytic anemia, disseminated intravascular coagulopathy, rhabdomyolysis, myonecrosis, renal failure, coma, and death which occurs in approximately 1% of confirmed bites [6].

Treatment

As the presentation varies from local effects to severe life-threatening complications so does the treatment for recluse envenomation. 

For patients with local effects the mainstay of treatment is wound care and pain management which can be as simple as wound observation, nonsteroidal anti-inflammatory drugs (NSAIDs), and possibly updating tetanus immunization. For recluse bites that have signs of developing necrosis, no proven therapy aside from administration of antivenom exists, however, antivenom therapy is not currently available in the United States [7] therefore in patients with dermal necrosis the mainstay of treatment remains with local wound care and pain management. 

For patients with systemic effects the treatment remains in providing supportive care such as transfusing red blood cells in patients that develop hemolytic anemia and trending hemoglobin/hematocrit [8]. In patients with rhabdomyolysis, rapid infusion of isotonic saline to establish urine output of 200 to 300 mL/hour (4 mL/kg per hour in children) with a goal of preventing renal failure. As patient illness severity increases or progresses, patients must be monitored for further and more invasive supportive therapy such as intubation and ventilation in patients that need respiratory support. 

When evaluating a patient with an isolated wound with unknown etiology loxoscelism remains on the differential diagnosis even though in the cases of simple wound management or pain control no specific therapy is being implemented that is unique to loxoscelism. However, in severe life-threatening cases of loxoscelism the treatment can be much more intensive from infusion of isotonic saline, packed red blood cell transfusion, correcting or treating electrolyte abnormalities, to much more intensive therapy if renal failure, encephalopathy, or respiratory failure progresses or persists. 

If loxoscelism is suspected, then further evaluation should be pursued. Recommended further laboratory evaluation includes complete blood count with peripheral smear and reticulocyte count; type and screen with Coombs; liver function panel; serum lactate dehydrogenase, haptoglobin, electrolytes, calcium, phosphate, and uric acid if signs of rhabdomyolysis, blood urea nitrogen and creatinine, creatine kinase; rapid urine dipstick for blood and for urobilinogen with reflex to urinalysis if positive; prothrombin time (PT) with international normalized ratio (INR), activated partial thromboplastin time (aPTT), and fibrinogen; and D-dimer; and electrocardiogram if rhabdomyolysis and electrolyte abnormalities are present [9,10]. 

The patient case presented the unique complication of loxoscelism of acute hemolytic anemia that required transfusion. Fortunately, the patient returned to her baseline health and required no further intervention and was stable for discharge to home. 

## Conclusions

When practicing medicine in the southern and mid-western United States and evaluating a patient with a single, isolated skin wound, loxoscelism should remain on the differential diagnosis and prompt further investigation, if suspected. Treatment varies from “do nothing” to “do everything” but overall treatment remains supportive as there is no immediate and specific directed therapy that changes disease course aside from management of complications of loxoscelism. 

The patient case that we presented is included in the 1% of envenomation causing life-threatening conditions. She suffered from an envenomation complication of acute hemolytic anemia that necessitated transfusion of blood after which she subsequently improved and was discharged home.

## Figures and Tables

**Figure 7:**
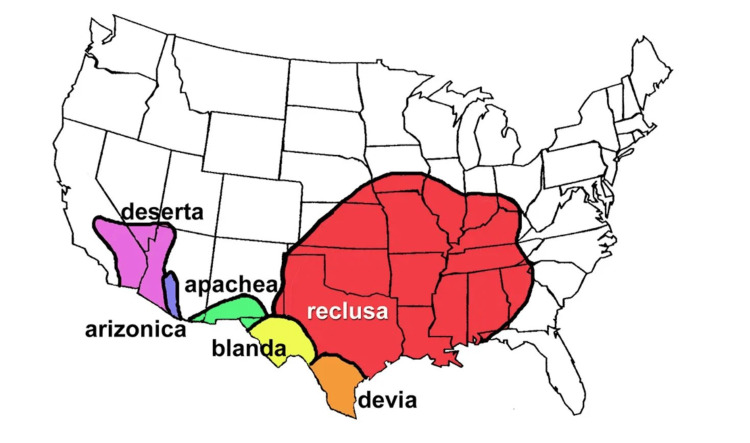
Distribution of recluse spiders in the United States (red area) This figure shows the distribution of recluse spiders in the United States showing multiple species. In red is the dominant recluse species in the southeastern area of the United States. Image graciously provided by the University of California Riverside, Department of Entomology and permission for use was obtained from the original publisher.

**Figure 8:**
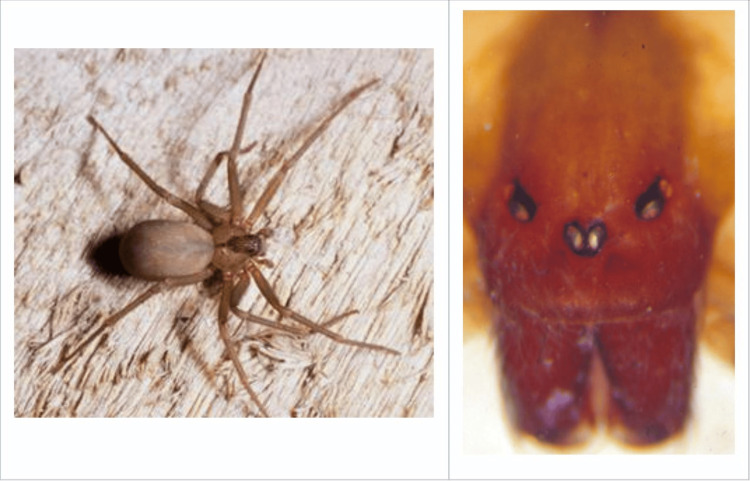
Identification of recluse spider This image shows an example of the two main methods for identification. The most accurate method of identifying a recluse spider involves counting the eyes if one can safely identify them by up-close examination. Recluse spiders have six eyes, with a pair in front, a pair on both sides, and a gap between the pairs. With the naked eye or low magnification, the eye pairs (dyads) may appear as individual eyespots. A less reliable method for identification is via body markings. The brown recluse has a violin pattern on its anterior cephalothorax, but this method is not as reliable. Image graciously provided by the University of California Riverside, Department of Entomology and permission for use was obtained from the original publisher.

## Appendices

Appendix A 

Appendix B 

## References

[1] Rick Vetter Staff Research Associate (2024). Brown Recluse spider map. Spider Research, UC Riverside 17 Sept 2005.

[2] Vetter RS, Barger DK (2002). An infestation of 2,055 brown recluse spiders (Araneae: Sicariidae) and no envenomations in a Kansas home: implications for bite diagnoses in nonendemic areas. J Med Entomol.

[3] Vetter Vetter, Richard Richard (2024). How to identify and misidentify a Brown Recluse spider. Spider Research, 17 Sept 2020.

[4] Anderson PC (1991). Loxoscelism threatening pregnancy: five cases. Am J Obstet Gynecol.

[5] Isbister GK, Fan HW (2011). Spider bite. Lancet.

[6] Anderson PC (1997). Spider bites in the United States. Dermatol Clin.

[7] Stahl WM (1987). Acute phase protein response to tissue injury. Crit Care Med.

[8] Streeper RT, Izbicka E (2022). Diethyl azelate for the treatment of Brown Recluse spider bite, a neglected orphan indication. In Vivo.

[9] Jacobs JW, Bastarache L, Thompson MA (2022). Laboratory predictors of hemolytic anemia in patients with systemic loxoscelism. Am J Clin Pathol.

[10] Swanson DL, Vetter RS (2006). Loxoscelism. Clin Dermatol.

